# Phenotype-genotype association grid: a convenient method for summarizing multiple association analyses

**DOI:** 10.1186/1471-2156-7-30

**Published:** 2006-05-22

**Authors:** Daniel Levy, Steven R DePalma, Emelia J Benjamin, Christopher J O'Donnell, Helen Parise, Joel N Hirschhorn, Ramachandran S Vasan, Seigo Izumo, Martin G Larson

**Affiliations:** 1 From the National Heart, Lung, and Blood Institute, Bethesda, MD, USA; 2 National Heart, Lung, and Blood Institute's Framingham Heart Study, Framingham, MA, USA; 3 Cardiology Division, Beth Israel-Deaconess Medical Center, Boston, MA, USA; 4 Division of Cardiology; 5 Department of Preventive Medicine, Boston University School of Medicine, Boston, MA, USA; 6 Department of Genetics, Harvard Medical School and Howard Hughes Medical Institute, Boston, MA, USA; 7 Division of Cardiology, Massachusetts General Hospital, Boston, MA, USA; 8 Department of Mathematics and Statistics, Boston University, Boston, MA, USA; 9 Divisions of Genetics and Endocrinology, Children's Hospital, Boston. MA, USA; 10 Broad Center at Harvard and MIT, Cambridge, MA, USA; 11 Novartis Research Institute, Cambridge, MA, USA

## Abstract

**Background:**

High-throughput genotyping generates vast amounts of data for analysis; results can be difficult to summarize succinctly. A single project may involve genotyping many genes with multiple variants per gene and analyzing each variant in relation to numerous phenotypes, using several genetic models and population subgroups. Hundreds of statistical tests may be performed for a single SNP, thereby complicating interpretation of results and inhibiting identification of patterns of association.

**Results:**

To facilitate visual display and summary of large numbers of association tests of genetic loci with multiple phenotypes, we developed a Phenotype-Genotype Association (PGA) grid display. A database-backed web server was used to create PGA grids from phenotypic and genotypic data (sample sizes, means and standard errors, P-value for association). HTML pages were generated using Tcl scripts on an AOLserver platform, using an Oracle database, and the ArsDigita Community System web toolkit. The grids are interactive and permit display of summary data for individual cells by a mouse click (i.e. least squares means for a given SNP and phenotype, specified genetic model and study sample). PGA grids can be used to visually summarize results of individual SNP associations, gene-environment associations, or haplotype associations.

**Conclusion:**

The PGA grid, which permits interactive exploration of large numbers of association test results, can serve as an easily adapted common and useful display format for large-scale genetic studies. Doing so would reduce the problem of publication bias, and would simplify the task of summarizing large-scale association studies.

## Background

The advent of high-throughput technology is generating unprecedented amounts of genotypic data that are being used in association analyses for multiple phenotypes. A single project may involve genotyping many genes with several variants (such as single nucleotide polymorphisms [SNPs]) per gene and analyzing each variant in relation to numerous phenotypes. In turn, each phenotype-SNP pair may be subjected to multiple genetic models and subgroup analyses. Hundreds of statistical tests may be performed for a single SNP, thereby complicating interpretation of results and inhibiting identification of patterns of association within a vast sea of data. Ultra-dense genome scans using 300,000 to 1,000,000 SNPs [1-3]will require efficient methods for analysis and presentation of results.

We are currently studying common SNPs in 200 candidate genes to test associations with alterations in echocardiographic phenotypes in participants from NHLBI's Framingham Heart Study. For each SNP, 144 statistical tests are performed: genotypes are analyzed with regard to six phenotypes (left ventricular [LV] mass, LV internal dimension, LV wall thickness, left atrial dimension, aortic dimension) through four genetic models (general, dominant, additive, recessive), with two levels of covariate adjustment (age and sex; age, sex and multiple additional covariates) in three samples (pooled sexes, men, women). Planned analyses of 1500 SNPs will generate nearly one quarter of a million statistical tests. Further details can be found on the CardioGenomics website [4].

As analyses commenced, it became obvious that we needed summary methods of data distillation and presentation to highlight findings of potential importance and to identify patterns of association, such as associations limited to one of multiple phenotypes, or associations limited to one sex. Therefore, we developed an approach that displays strengths of statistical associations at a glance, and that makes supporting data available easily via graphs accessed by a mouse click.

## Results

**Figure 1:**
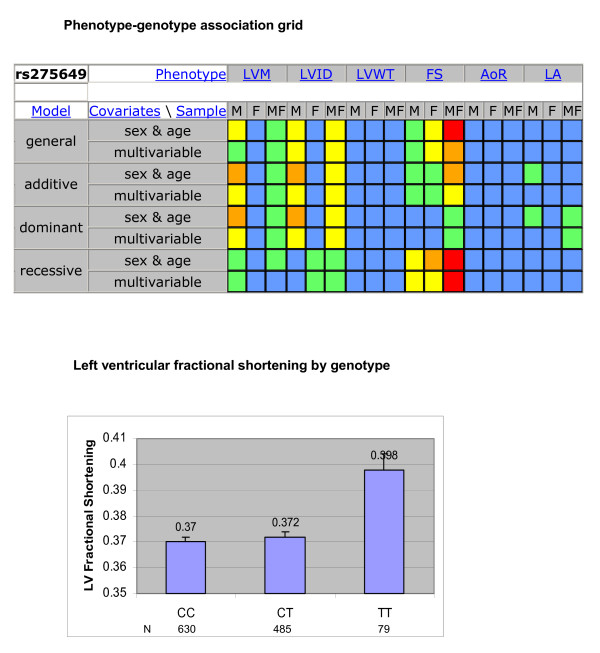
**Phenotype-genotype association (PGA) grid**. Top Panel: Phenotype-Genotype Association Grip for SNP rs275649. 144 tests of association are displayed in color-coded cells. Colors indicate level of statistical significance: blue p ≥ 0.05, green 0.01 ≤ P < 0.05, yellow 0.001 ≤ P < 0.01, orange 0.0001 ≤ P < 0.001, red P < 0.0001. (Tests with fewer than 10 participants for a genotype are identified by an asterisk to alert the user that the estimates may be unstable. That was not the case for this example.) Bottom Panel: Least Squares Means Plot of Left Ventricular Fractional Shortening by Genotype Mean values (and standard errors) for left ventricular fractional shortening, by genotype for SNP rs275649, based on a general model that adjusted for age and sex in the pooled sample of men and women (p = 3.8 × 10^5^). (Tests with fewer than 10 participants for a genotype are identified by cross-hatching of corresponding bars to alert the user that the estimates may be unstable. That was not the case for this example.)

Figure 1 (top panel) presents a Phenotype-Genotype Association (PGA) grid for a single SNP. Color coding denotes levels of statistical significance. In this example, associations having nominal P-values <0.05 were observed for four of six phenotypes and patterns of significance differed by sex. The color/visual aspect of the grid also helps in discerning patterns of association among related phenotypes.

The PGA grid is interactive. Clicking on a specific cell generates a plot of adjusted least squares means for the trait of interest by genotype for the corresponding genetic model. Figure 1 (bottom panel) displays this plot for the highlighted cell in Figure 1 (LV fractional shortening for pooled sexes, general model, adjusted for age and sex). At the gene level, thumbnail PGA grids for each typed SNP are displayed on a single page with each thumbnail sorted by map position and hyperlinked to its full-sized parent grid. The underlying database can be searched by gene, P-value, or phenotype to facilitate hypothesis generation and pursuit [4].

**Figure 2:**
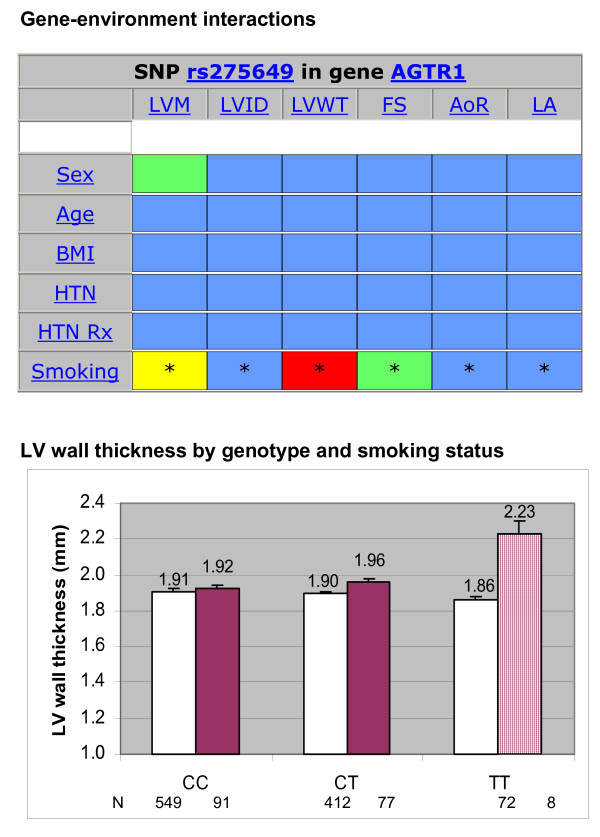
**Gene-environment interaction**. Top Panel: Gene environment interactions for SNP rs275649. Interaction test results for the six phenotypes by are presented for sex (men vs. women), age, body mass index, hypertension (yes vs. no), hypertension treatment (yes vs. no) and cigarette smoking (yes vs. no). Color coding of statistical significance levels is the same as presented in Figure 1. Asterisks designate cells with fewer than 10 observations in one of the phenotype-genotype subgroups. Bottom Panel: Mean values (and standard errors) for left ventricular wall thickness (LVWT) for SNP rs275649 by genotype and cigarette smoking status (nonsmokers in open bars, smokers in filled bars). Test for interaction yielded p < 0.0001. Data are adjusted for age and sex and clinical covariates. The cross hatched bar indicates a group with fewer than 10 subjects.

**Figure 3:**
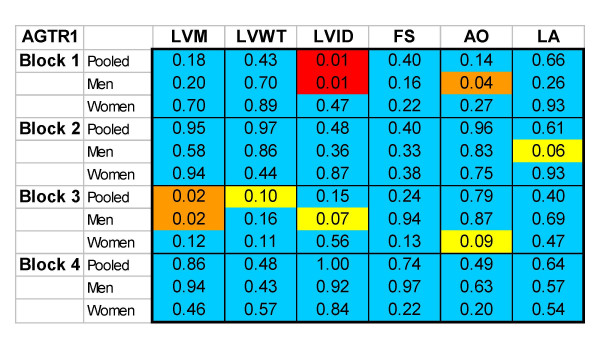
**Haplotype-block association grid**. This figure displays block-specific haplotype associations with six phenotypes. P-values are based on a global test of differences in phenotype across all haplotypes within the block. Color coding reflects a global test of significance for differences among all haplotypes within a block (blue p ≥ 0.10, orange 0.05 ≤ P < 0.10, yellow ≤ 0.01 < P0.05, red P < 0.01).

The software to create PGA grids from user-supplied data (sample sizes, means and standard errors, P-value for association) utilizes a database-backed web server. We generate HTML pages using Tcl scripts on an AOLserver platform [5] using an Oracle database [6], and the ArsDigita Community System web toolkit [7]. Source code (see Additional Files 1 and 2, available upon request) is available for free download [8]and can be adapted for use elsewhere on other database-backed web platforms. The grid can be modified to display results for gene-environment interactions (Figure 2). In addition the grid can used to summarize analyses of qualitative traits or haplotypes [3]. For example, one could display a grid for each gene with cells to indicate block-specific P-values based on a global test of differences in phenotype across all haplotypes within the block (Figure 3).

## Discussion

The PGA grid was developed to summarize large numbers of phenotype-genotype association tests in a visually useful manner to facilitate interactive exploration of results. This approach could serve as a common format for large-scale association studies. Due to the large number of association tests performed, there will be many nominally significant results. One approach to multiple testing is to indicate P-values deemed statistically significant based on consideration of false discovery rates [9,10]. Most association tests, however, will yield results that do not achieve significance on their own, but that are valuable in the context of other studies of the same gene [11]. Unfortunately, in most large-scale association studies negative or inconclusive results are usually suppressed during publication or at best presented in extremely abridged form.

## Conclusion

The PGA grid provides a simple visual method for displaying a large number of results, potentially reduces the problem of publication bias, and simplifies the task of summarizing large-scale association studies.

## Abbreviations

LVM = left ventricular mass; LVID = left ventricular internal diameter at end diastole; LVWT = sum of septal and left ventricular posterior wall thickness; FS = left ventricular fractional shortening; AoR = aortic root diameter; LA = left atrial anteroposterior dimension. M = men only; F = women only; MF = men and women.

## Authors' contributions

Daniel Levy: Conception of the display grid, drafting of paper, revisions to manuscript

Steven R. DePalma: Development of source code for display grid, revisions to manuscript

Emelia J. Benjamin: Conception of the display grid, revisions to manuscript

Christopher J. O'Donnell: Conception of the display grid, revisions to manuscript

Helen Parise: Statistical analyses for incorporation into display grid

Joel N. Hirschhorn: Conception of the display grid, revisions to manuscript

Ramachandran S. Vasan: Conception of the display grid, revisions to manuscript

Seigo Izumo: Principal investigator of CardioGenomics, funding of the project

Martin G. Larson: Conception of the display grid, development of statistical methods, revisions to manuscript

## Supplementary Material

Additional File 1 "pga-grid-v1.01-src.zip" is the source code for PGA Grid, version 1.01, as a .zip archive containing 31 text files (.tcl, .sql, .pl, .js, .css, .htm, .txt) for use with a Linux/AOLserver/Oracle/ACS web server platform. File descriptions are available in Additional File 2, pga-grid-v1.01-readme.htm. The most recent version of this software is available from . Click here for file

Additional File 2 "pga-grid-v1.01-readme.htm" is an HTML-format file that lists and describes each of the files contained in Additional File 1, pga-grid-v1.01-src.zip. Click here for file
