# Supplementary material for: Phenotype-genotype association grid: a convenient method for summarizing multiple association analyses
Source: BMC Genet. 2006 May 22;7:30. doi: 10.1186/1471-2156-7-30 (PMC1526453; doi:10.1186/1471-2156-7-30)
Supplement: Additional File 1 — "pga-grid-v1.01-src.zip" is the source code for PGA Grid, version 1.01, as a .zip archive containing 31 text files (.tcl, .sql, .pl, .js, .css, .htm, .txt) for use with a Linux/AOLserver/Oracle/ACS web server platform. File descriptions are available in Additional File 2, pga-grid-v1.01-readme.htm. The most recent version of this software is available from . [file 1471-2156-7-30-S1.htm]

home 
Source code for PGA Grid, ver. 1.01
PGA Grid is an HTML-based system created for displaying results of
large-scale \*P\*henotype-\*G\*enotype \*A\*ssociation studies. It was
developed under the auspices of NHLBI's CardioGenomics Program for
Genomic Applications .
Each grid packages the results of a large number of association tests in
such a way that strengths of associations are interpretable at a glance,
while at the same time making the supporting data available easily in
the form of graphs that can be accessed at the click of a mouse.
The source code is written for our platform of a Red Hat Linux machine
running AOLserver  v3.4.2, Oracle
 8i, and the ArsDigita Community System
 web toolkit,
v3.2.0 (docs ). Graphics-drawing tools required are
Thomas Boutell's GD  (we use Spencer
Thomas's Tcl interface to GD, gdtclft ), and
ChartDirector .
Version 1.01 of PGA Grid was customized to the particular requirements
of the first CardioGenomics association project (between left
ventricular echocardiographic phenotype and SNP genotype). As such, this
source code is provided primarily as a reference rather than as a
standalone software application. Future versions will be more
generalized to allow flexibility in choice of parameters. The most
recent version of this software can be found at
http://cardiogenomics.med.harvard.edu/src/pga-grid/.
Note that association statistics are calculated beforehand; these pages
only display precalculated numbers. The file
pg-input-sample-primary1.txt is a tab-delimited text file containing a
simulated sample of the input data. This sample file is processed by a
Perl script, process-pvalue-spreadsheet-sql.pl
, to create .sql files to update
database tables.
------------------------------------------------------------------------
The files
assoc-results.tcl 
Presents display choices for viewer: Display gene-list, display all
thumbnails, or search for low pvalues. A good place to start
viewing. /(view source )/
assoc-gene.tcl 
Displays PGA Grid thumbnails for all SNPs in a given gene. (/view
source /)
assoc-search-2.tcl 
Displays results of search-by-pvalue, with grid thumbnails. (/view
source /)
assoc-all-icons.tcl 
Displays all Primary PGA Grid thumbnails (icons) in all genes, on
one page. (/view source /)
gei-all-icons.tcl 
Displays all Gene-Environment Interaction grid thumbnails in all
genes, on one page. (/view source /)
assoc-grid-frame.tcl 
Frameset for assoc-grid.tcl and lsm-graph.tcl. (/view source
/)
gei-grid-frame.tcl 
Frameset for gei-grid.tcl and gei-graph.tcl. (/view source
/)
assoc-grid.tcl 
Creates Primary PGA Grid as HTML table, with hyperlinks to bar
graphs of supporting data. Top frame in a frameset. (/view source
/)
gei-grid.tcl 
Creates PGA Grid as HTML table, for Gene-Environment Interaction
associations. Top frame in a frameset. (/view source
/)
lsm-graph.tcl
Creates and displays bar graph in frame under assoc-grid.tcl. Bottom
frame in a frameset. (/view source /)
gei-graph.tcl 
Displays bar graph in frame under gei-grid.tcl. Bottom frame in a
frameset. (/view source /)
cgi-bin/gei-graph.pl
Perl CGI script that creates bar graphs which are called by and
displayed by gei-graph.tcl. (/view source /)
stats/assoc-gene-list.tcl 
Ranks genes by a significance metric, to identify genes with the
most interesting p-values. (/view source /)
stats/pv-dist.tcl
Displays graph and table showing distribution of p-values for SNPs
in a gene, according to various user-set criteria. (/view source
/)
cgi-bin/pv-dist.pl
CGI script that uses ChartDirector to draw a graph for pv-dist.tcl.
(/view source /)
tcl/p5-defs.tcl
Tcl procedures to support Project 5 (i.e. Primary PGA Grid) pages.
(/view source /)
tcl/p5-graph-defs.tcl
Tcl procedures to help generate bar graphs. (/view source
/)
tcl/p5-gei-defs.tcl
Tcl procedures to support GEI grid pages. (/view source
/)
sql/cg.sql
Common CardioGenomics Oracle tables (e.g., cg\_genes). (/view source
/)
sql/cg-funcs.sql
PL/SQL functions to support database queries (e.g., cg\_chrom\_order)
(/view source /)
sql/p5-assoc.sql
Oracle tables specific to PGA Grid (Project 5). (/view source
/)
sql/p5-funcs.sql
PL/SQL functions to support PGA Grid queries. (/view source
/)
p5.css
Cascading Style Sheet for PGA Grid pages. (/view source /)
p5.js
Javascript functions for PGA Grid pages. (/view source /)
one-snp-hw.tcl 
Displays Hardy-Weinberg Equilibrium data for one SNP. (/view source
/)
icon-test-utility.tcl 
Developer's utility that creates random-value PGA Grid thumbnails in
configurable sizes. Try it. (/view source /)
------------------------------------------------------------------------
View the CardioGenomics warranty disclaimer and copyright notice
. The PGA Grid software package is distributed under the
GNU General Public License . It is
open source software which you are free to use and modify, as long as
any redistributions you may make are also covered by the GNU GPL.
Author: Steve DePalma, depalma@rascal.med.harvard.edu, 5-Dec-2005
