# Supplementary material for: Phenotype-genotype association grid: a convenient method for summarizing multiple association analyses
Source: BMC Genet. 2006 May 22;7:30. doi: 10.1186/1471-2156-7-30 (PMC1526453; doi:10.1186/1471-2156-7-30)
Supplement: Additional File 2 — "pga-grid-v1.01-readme.htm" is an HTML-format file that lists and describes each of the files contained in Additional File 1, pga-grid-v1.01-src.zip. [file 1471-2156-7-30-S2.zip › pga-grid-v1.01/pga-grid-v1.01-readme.htm]

Source code for PGA Grid, ver. 1.01


## Source code for PGA Grid, ver. 1.01

PGA Grid is an HTML-based system created for displaying results
of large-scale **P**henotype-**G**enotype **A**ssociation studies.
It was developed under the auspices of NHLBI's CardioGenomics Program for Genomic Applications. Each grid packages the results
of a large number of association tests in such a way that strengths of
associations are interpretable at a glance, while at the same time
making the supporting data available easily in the form of graphs that
can be accessed at the click of a mouse.

The source code is written for our platform of a Red Hat Linux
machine running AOLserver v3.4.2, Oracle 8i, and the ArsDigita Community System web toolkit, v3.2.0
(docs).
Graphics-drawing
tools required are Thomas Boutell's GD (we use Spencer Thomas's Tcl interface to GD,
gdtclft), and
ChartDirector.

Version 1.01 of PGA Grid was customized to the particular
requirements of the first CardioGenomics association project (between left
ventricular echocardiographic phenotype and SNP genotype). As such,
this source code is provided primarily as a reference rather than as a
standalone software application. Future versions
will be more generalized to allow flexibility in choice of
parameters. The most recent version of this software can be found at
http://cardiogenomics.med.harvard.edu/src/pga-grid/.

Note that association statistics are calculated beforehand; these
pages only display precalculated numbers. The file
pg-input-sample-primary1.txt
is a tab-delimited text file containing a simulated sample of the input data.
This sample file is processed by a Perl script,
process-pvalue-spreadsheet-sql.pl, to create .sql files to update
database tables.

---

### The files

assoc-results.tcl
:   Presents display choices for viewer: Display gene-list, display
    all thumbnails, or search for low pvalues. A good place to start viewing.
    *(view source)*

assoc-gene.tcl
:   Displays PGA Grid thumbnails for all SNPs in a given gene.
    (*view source*)

assoc-search-2.tcl
:   Displays results of search-by-pvalue, with grid thumbnails.
    (*view source*)

assoc-all-icons.tcl
:   Displays all Primary PGA Grid thumbnails (icons) in all genes, on one page.
    (*view source*)

gei-all-icons.tcl
:   Displays all Gene-Environment Interaction grid thumbnails in all
    genes, on one page.
    (*view source*)

assoc-grid-frame.tcl
:   Frameset for assoc-grid.tcl and lsm-graph.tcl.
    (*view source*)

gei-grid-frame.tcl
:   Frameset for gei-grid.tcl and gei-graph.tcl.
    (*view source*)

assoc-grid.tcl
:   Creates Primary PGA Grid as HTML table, with hyperlinks to bar graphs of supporting data. Top frame in a frameset.
    (*view source*)

gei-grid.tcl
:   Creates PGA Grid as HTML table, for Gene-Environment Interaction associations. Top frame in a frameset. (*view source*)

lsm-graph.tcl
:   Creates and displays bar graph in frame under assoc-grid.tcl. Bottom frame in a frameset.
    (*view source*)

gei-graph.tcl
:   Displays bar graph in frame under gei-grid.tcl. Bottom frame in a frameset.
    (*view source*)

cgi-bin/gei-graph.pl
:   Perl CGI script that creates bar graphs which are called by and displayed by gei-graph.tcl.
    (*view source*)

stats/assoc-gene-list.tcl
:   Ranks genes by a significance metric, to identify genes with the most
    interesting p-values.
    (*view source*)

stats/pv-dist.tcl
:   Displays graph and table showing distribution of p-values for SNPs
    in a gene, according to various user-set criteria.
    (*view source*)

cgi-bin/pv-dist.pl
:   CGI script that uses ChartDirector to draw a graph for pv-dist.tcl.
    (*view source*)

tcl/p5-defs.tcl
:   Tcl procedures to support Project 5 (i.e. Primary PGA Grid) pages.
    (*view source*)

tcl/p5-graph-defs.tcl
:   Tcl procedures to help generate bar graphs.
    (*view source*)

tcl/p5-gei-defs.tcl
:   Tcl procedures to support GEI grid pages.
    (*view source*)

sql/cg.sql
:   Common CardioGenomics Oracle tables (e.g., cg\_genes).
    (*view source*)

sql/cg-funcs.sql
:   PL/SQL functions to support database queries (e.g., cg\_chrom\_order)
    (*view source*)

sql/p5-assoc.sql
:   Oracle tables specific to PGA Grid (Project 5).
    (*view source*)

sql/p5-funcs.sql
:   PL/SQL functions to support PGA Grid queries.
    (*view source*)

p5.css
:   Cascading Style Sheet for PGA Grid pages. (*view source*)

p5.js
:   Javascript functions for PGA Grid pages.
    (*view source*)

one-snp-hw.tcl
:   Displays Hardy-Weinberg Equilibrium data for one SNP.
    (*view source*)

icon-test-utility.tcl
:   Developer's utility that creates random-value PGA Grid thumbnails in
    configurable sizes. Try it.
    (*view source*)

---

View the CardioGenomics warranty disclaimer and copyright notice. The PGA Grid software package is distributed under the
GNU General Public License.
It is open source software which you are free to use and modify, as long as
any redistributions you may make are also covered by the GNU GPL.
Author: Steve DePalma, depalma@rascal.med.harvard.edu, 5-Dec-2005
